# Fluoride Depletes Acidogenic Taxa in Oral but Not Gut Microbial Communities in Mice

**DOI:** 10.1128/mSystems.00047-17

**Published:** 2017-08-08

**Authors:** Koji Yasuda, Tiffany Hsu, Carey A. Gallini, Lauren J. Mclver, Emma Schwager, Andy Shi, Casey R. DuLong, Randall N. Schwager, Galeb S. Abu-Ali, Eric A. Franzosa, Wendy S. Garrett, Curtis Huttenhower, Xochitl C. Morgan

**Affiliations:** a Department of Biostatistics, Harvard T.H. Chan School of Public Health, Boston, Massachusetts, USA; b Broad Institute of MIT and Harvard, Cambridge, Massachusetts, USA; c Department of Immunology and Infectious Diseases, Harvard T.H. Chan School of Public Health, Boston, Massachusetts, USA; d Department of Genetics and Complex Diseases, Harvard T.H. Chan School of Public Health, Boston, Massachusetts, USA; e Department of Medical Oncology, Dana-Farber Cancer Institute, Boston, Massachusetts, USA; f Department of Microbiology and Immunology, The University of Otago, Dunedin, New Zealand; Qingdao Institute of Bioenergy and Bioprocess Technology, Chinese Academy of Sciences

**Keywords:** 16S rRNA sequencing, fluoridation, fluoride, gut microbiome, mouse, oral microbiome, shotgun metagenomic sequencing

## Abstract

Fluoridation of drinking water and dental products prevents dental caries primarily by inhibiting energy harvest in oral cariogenic bacteria (such as *Streptococcus mutans* and *Streptococcus sanguinis*), thus leading to their depletion. However, the extent to which oral and gut microbial communities are affected by host fluoride exposure has been underexplored. In this study, we modeled human fluoride exposures to municipal water and dental products by treating mice with low or high levels of fluoride over a 12-week period. We then used 16S rRNA gene amplicon and shotgun metagenomic sequencing to assess fluoride’s effects on oral and gut microbiome composition and function. In both the low- and high-fluoride groups, several operational taxonomic units (OTUs) belonging to acidogenic bacterial genera (such as *Parabacteroides*, *Bacteroides*, and *Bilophila*) were depleted in the oral community. In addition, fluoride-associated changes in oral community composition resulted in depletion of gene families involved in central carbon metabolism and energy harvest (2-oxoglutarate ferredoxin oxidoreductase, succinate dehydrogenase, and the glyoxylate cycle). In contrast, fluoride treatment did not induce a significant shift in gut microbial community composition or function in our mouse model, possibly due to absorption in the upper gastrointestinal tract. Fluoride-associated perturbations thus appeared to have a selective effect on the composition of the oral but not gut microbial community in mice. Future studies will be necessary to understand possible implications of fluoride exposure for the human microbiome.

**IMPORTANCE** Fluoride has been added to drinking water and dental products since the 1950s. The beneficial effects of fluoride on oral health are due to its ability to inhibit the growth of bacteria that cause dental caries. Despite widespread human consumption of fluoride, there have been only two studies of humans that considered the effect of fluoride on human-associated microbial communities, which are increasingly understood to play important roles in health and disease. Notably, neither of these studies included a true cross-sectional control lacking fluoride exposure, as study subjects continued baseline fluoride treatment in their daily dental hygiene routines. To our knowledge, this work (in mice) is the first controlled study to assess the independent effects of fluoride exposure on the oral and gut microbial communities. Investigating how fluoride interacts with host-associated microbial communities in this controlled setting represents an effort toward understanding how common environmental exposures may potentially influence health.

## Abstract (precis)

Fluoride has been added to drinking water and dental products since the 1950s. The beneficial effects of fluoride on oral health are due to its ability to inhibit the growth of bacteria that cause dental caries. Despite widespread human consumption of fluoride, there have been only two studies of humans that considered the effect of fluoride on human-associated microbial communities, which are increasingly understood to play important roles in health and disease. Notably, neither of these studies included a true cross-sectional control lacking fluoride exposure, as study subjects continued baseline fluoride treatment in their daily dental hygiene routines. To our knowledge, this work (in mice) is the first controlled study to assess the independent effects of fluoride exposure on the oral and gut microbial communities. Investigating how fluoride interacts with host-associated microbial communities in this controlled setting represents an effort toward understanding how common environmental exposures may potentially influence health.

## INTRODUCTION

Since the 1940s, fluoridation of drinking water and dental products has been employed as a public health measure to prevent dental caries. In the United States, more than 60% of municipal water is fluoridated, and the majority of dental products contain fluoride (1–3). Fluoridated compounds improve oral health by inhibiting bacterial growth through inhibition of the enzyme enolase, which catalyzes the conversion of 2-phosphoglycerate to phosphoenolpyruvate (the last step of anaerobic glycolysis) and thus is critical for microbial energy harvest and growth (4, 5). Inhibition of individual oral bacteria such as *Streptococcus mutans* by fluoride has been well studied (6–8), but how fluoride affects the overall oral microbiome or the gut microbiome has been underinvestigated.

The major mechanisms by which fluoride inhibits bacterial energy growth are direct binding of the fluorine ion to the active sites of enolase (5) and ATPases (9) and disruption of the ion gradient across the bacterial cell membrane (10, 11). All of these mechanisms result in the reduction of ATP synthesis (9). Although a wide range of bacterial taxa are inhibited by this mechanism, the degree of resistance differs across taxa. For example, the enolases of* Streptococcus mutans* and* Streptococcus sanguis* are more susceptible (by 10-fold) to fluoride than those of *Streptococcus salivarius* and *Lactobacillus casei* in monoculture (9, 12). This variation in fluoride resistance raises the question of how fluoride affects individual bacterial taxa within a complex microbial community. Two recent studies have begun extending this line of investigation to dental plaque microbial communities using high-throughput sequencing (13, 14). Unfortunately, neither of these studies included a true control group to properly assess fluoride’s effects on the oral microbiome (individuals in the studies’ control groups retained access to commercial fluoride-containing dental products and fluoride in drinking water during the experimental period). Moreover, one of these studies considered a single dose of sodium fluoride mouthwash (13) and was therefore not designed to assess the effects of longer-term exposures. Thus, no existing study has tested the effects of fluoride exposures at the levels found in municipal water and dental products on the oral and gut microbial communities.

We address these questions by assessing oral and stool microbiome structures and their functional potentials in mice given (i) nonfluoridated drinking water, (ii) fluoridated drinking water, or (iii) daily fluoride gavage in addition to fluoridated drinking water over 12 weeks. 16S rRNA gene amplicon and shotgun metagenomic sequencing revealed that fluoride exposure significantly perturbed oral but not gut microbiome composition in mice. Specifically, fluoride selectively depleted oral acidogenic bacteria, including *Bacteroides*, *Parabacteroides*, and *Bilophila*. In terms of the microbial functional profiles, fluoride exposure selectively depleted metabolic modules important for central carbon metabolism. Our results support the idea that fluoride-associated perturbations have a selective effect on the composition of the oral microbial community in mice. Though limited by lack of human data, this study suggests that the levels of fluoride currently added to drinking water and associated with routine use of dental products are unlikely to have significant effects on established gut microbial communities.

## RESULTS

### Drivers of oral and gut microbial diversity in fluoride-treated mice.

To elucidate the effects of chronic fluoride exposure on oral and gut microbial communities, wild-type BALB/c mice (1 month of age) were randomized upon weaning to the following experimental groups: (i) unfluoridated (deionized) drinking water, (ii) fluoridated drinking water (4 ppm), or (iii) fluoridated drinking water (4 ppm) plus a daily gavage of fluoride similar to a dose ingested when swallowing dental fluoride products (2.25 µg of fluoride per day via gavage) for a period of 12 weeks. Oral samples were collected at 0 and 12 weeks, and stool samples were collected at 0, 4, 8, and 12 weeks. Since the collection of oral samples required animals to be sacrificed, eight mice were taken for oral week 0 sample at random when the rest of the animals were allocated to the study groups. To measure the effects of fluoride on the composition of the microbial community, we used amplicon sequencing of the 16S ribosomal rRNA gene, specifically hypervariable region V4 (referred to hereafter as 16S) (see Materials and Methods).

**FIG 1:**
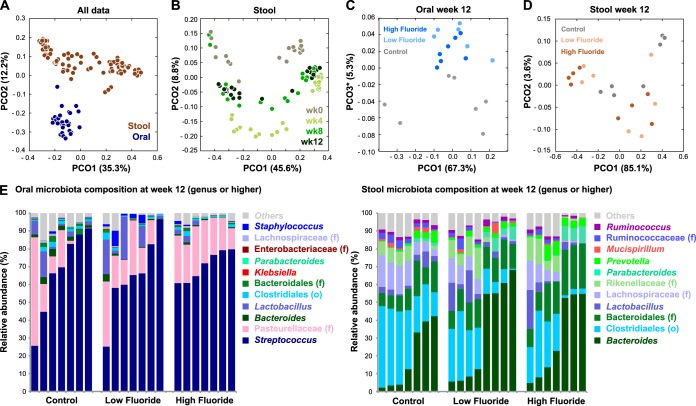
Drivers of oral and gut microbial diversity in fluoride-treated mice. (A) Principal-coordinate analysis (PCoA) of all samples by weighted UniFrac distance. PCO1, principal coordinate 1. (B) PCoA of stool samples by weighted UniFrac distance. (C) PCoA of oral samples at week 12 by weighted UniFrac distance. (D) PCoA of stool samples at week 12 by weighted UniFrac distance. (E) Genus-level relative abundance of the oral and stool microbiota. Individual columns represent individual animals and are grouped by treatment (control, low, and high fluoride).

We first examined the major factors driving microbial diversity across our data set by applying permutational multivariate analysis of variance (PERMANOVA) (adonis R package) to weighted UniFrac distance. Biogeographical site (oral versus gut) explained the largest fraction of between-sample diversity (45%, *P* < 0.001; Fig. 1A). This observation is consistent with the strong effect of biogeography on microbial community structure seen in other mammals (15, 16). Downstream nonparametric comparison of oral and gut samples (17) revealed statistically significant enrichments of *Streptococcus* spp. and *Pasteurellaceae* species in the oral site, while the gut was enriched for *Bacteroides*, *Clostridiales*, *Lachnospiraceae*, and *Parabacteroides* (Fig. 1E and see Table S1 in the supplemental material). Notably, these clades are similarly enriched in human oral and gut sites, respectively (15, 18, 19).

10.1128/mSystems.00047-17.5 TABLE S1  Differentially abundant bacterial genera inferred from univariate analysis by oral-stool biogeography in mice. Download TABLE S1, XLS file, 0.02 MB. Copyright © 2017 Yasuda et al. 2017 Yasuda et al. This content is distributed under the terms of the Creative Commons Attribution 4.0 International license.

Housing cage (Fig. S1A) and treatment time point (Fig. 1B and Fig. S1B) explained additional, significant fractions of between-sample diversity (29% and 4%, respectively). Specifically, within-cage stool communities (Fig. S1A and E) were significantly more similar than between-cage communities (*P* < 0.001 by Mann-Whitney U test on weighted UniFrac distance), as observed in previous studies (20). These cage effects corresponded to recognizable sample subgroups dominated by either (i) *Clostridiales* and *Lachnospiraceae* or (ii) *Bacteroides* and *Parabacteroides*. Curiously, we did not find oral samples to be significantly more similar within cages than between cages (*P* = 0.34), suggesting that cage effects may be less pronounced among the mouse oral microbiota. On the basis of these observations, we explicitly controlled for cage effects in downstream analyses of stool.

10.1128/mSystems.00047-17.1 FIG S1  Drivers of oral and gut microbial diversity in fluoride-treated mice. (A) Principal-coordinate analysis (PCoA) of stool samples from all time points by weighted UniFrac distances colored by cages. (B) PCoA of week 12 oral samples by weighted UniFrac distance. (C) PCoA of week 12 stool by weighted UniFrac distances colored by cages. (D) PCoA of stool samples from all time points by weighted UniFrac distances colored by fluoride treatment groups. (E) Microbial community dissimilarity of oral and stool sites measured by weighted UniFrac distanced among mice within cages (cage mates [intracage]) and non-cage-mates (intercage). (F) Genus-level or higher relative abundance of the oral microbial compositions at week zero. (G) Genus-level or higher stool microbiota composition stratified by fluoride group and time. Download FIG S1, PDF file, 1.2 MB. Copyright © 2017 Yasuda et al. 2017 Yasuda et al. This content is distributed under the terms of the Creative Commons Attribution 4.0 International license.

While overview ordination suggested a slight separation among week 12 oral samples associated with fluoride treatment (Fig. 1C), we did not observe a separation in stool samples (Fig. 1D and Fig. S1D), and the component of between-sample diversity explained by fluoride was not statistically significant by PERMANOVA (*P* = 0.860). This suggests that fluoride treatment does not have a large effect on the global structure of the oral or gut microbiota in mice. However, it remained possible that fluoride treatment could selectively alter the abundance of individual microbial taxa and functions in these environments.

### Fluoride selectively depletes oral acidogenic taxa in mice.

**FIG 2:**
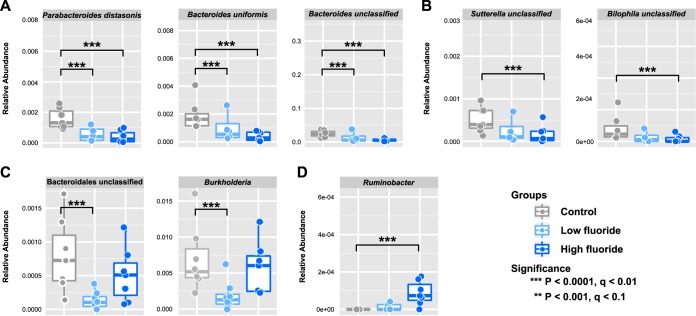
Fluoride selectively depletes acidogenic anaerobes in the oral microbiota. (A to C) Multivariate linear model association results (21) showing bacterial OTUs that are consistently depleted in low- and high-fluoride treatment groups (A), affected only in the high-fluoride group (B), or the low-fluoride group compared to the control group (C). (D) A bacterial OTU enriched in the high-fluoride group.

To identify bacterial taxa selectively affected by fluoride in the oral site, we performed multivariate analyses using MaAsLin (Multivariate Associations by Linear models) (21) with fluoride exposure coded as a categorical variable: high-fluoride exposure versus low-fluoride exposure versus no exposure (control). We considered only the week 12 oral samples (*n* = 21) and isolated associations with false-discovery rate (FDR)-corrected *q* values of <0.2 as statistically significant. In both the low- and high-fluoride groups, obligate anaerobes such as *Parabacteroides distasonis*, *Bacteroides uniformis*, and an unclassified species of *Bacteroides* were consistently depleted compared to the control group (Fig. 2A and Table S2). *Sutterella* and *Bilophila* were also depleted in fluoride-treated animals but only significantly so in the high-fluoride group (Fig. 2B). Such a pattern is consistent with previous *in vitro* studies of dosage-dependent inhibition of enolase in different microbial oral isolates (22). Curiously, unclassified species of *Bacteroidales* and *Burkholderia* were significantly depleted only in the low-fluoride group compared to the control group (Fig. 2C), possibly due to more extreme depletion of other taxa in the high-fluoride group. A similar mechanism would explain the expansion of another obligate anaerobe (an unclassified *Ruminobacter* species) in the high-fluoride group relative to the control group (Fig. 2D).

10.1128/mSystems.00047-17.6 TABLE S2  Fluoride treatment effects on oral bacterial OTUs at week 12. Download TABLE S2, XLS file, 0.02 MB. Copyright © 2017 Yasuda et al. 2017 Yasuda et al. This content is distributed under the terms of the Creative Commons Attribution 4.0 International license.

We performed a similar multivariate analysis to identify fluoride-sensitive taxa among stool samples. Relative to the model described above, we also considered treatment time point and animal cage as covariates (i.e., week 4, week 8, and week 12 samples were all considered). However, no species- or genus-level taxa showed significant (FDR-corrected *q* value of <0.2) associations with the high- or low-fluoride groups relative to the control group. Results were similar when applying the model separately to samples stratified by treatment time point. While our inability to detect a significant treatment effect in stool could be a result of small sample size, an alternative explanation is that orally administered fluoride does not reach the gut in large enough quantities to perturb the gut microbiota. Previous research has suggested that fluoride absorption occurs mainly in the stomach and upper small intestine (23). We confirmed this result by directly measuring fluoride levels in the stools of treated mice versus untreated mice. Even after 12 weeks of high-fluoride treatment, fluoride levels in the stools of treated mice were not significantly higher than baseline (control) stool fluoride levels (*P* > 0.05 by one-tailed *t* test; see Materials and Methods and Fig. S2). Hence, while the gut microbiota may be sensitive to fluoride, orally administered fluoride (even at high doses) is unlikely to expose this sensitivity.

10.1128/mSystems.00047-17.2 FIG S2  Measuring fluoride concentrations. Using a precalibrated fluoride ion probe, we measured fluoride concentrations in (i) deionized water, (ii) municipal water sampled at the Harvard T.H. Chan School of Public Health (Boston, MA), and (iii) mouse stool samples (100 mg in 10 ml of deionized water) from each treatment group at week 0 and week 12. Posttreatment stool fluoride concentrations were not significantly higher than baseline levels (*P* > 0.05 in all comparisons by one-tailed *t* test). A separate calibration series confirmed that the probe was able to detect the presence of added fluoride in the presence of stool with bead beating (blue) and without bead beating (green) (bead beating used to potentially release additional intracellular fluoride). Added fluoride could not be distinguished below baseline stool concentrations (~0.015 µg/µl of dissolved stool). Download FIG S2, PDF file, 0.03 MB. Copyright © 2017 Yasuda et al. 2017 Yasuda et al. This content is distributed under the terms of the Creative Commons Attribution 4.0 International license.

### Fluoride perturbs predicted oral microbial community functional potential.

In addition to affecting individual microbial taxa, fluoride exposure may alter community-level function by enriching or depleting taxa that encode specific metabolic modules. Notably, this could be true in the gut, even though individual taxa failed to show a significant effect there. To test this hypothesis in our data, we used PICRUSt (Phylogenetic Investigation of Communities by Reconstruction of Unobserved States) (24) to infer community gene content from 16S amplicon sequencing data, followed by HUMAnN (25) to reconstruct functional modules. We then applied the same multivariate testing framework described above to identify modules whose relative abundance varied with fluoride treatment (focusing on modules with relative abundance of >0.001% in at least five samples).

No functional modules varied significantly with fluoride treatment in the gut after adjusting for treatment time point and animal cage effects (all FDR-corrected *q* values of >0.2). However, in the oral samples, 19 of the 113 observed functional modules were differentially abundant among the treatment groups (Table S3). Fluoride treatment was associated with depletions in the glyoxylate cycle (Kyoto Encyclopedia of Genes and Genomes [KEGG] module M00012), succinate dehydrogenase (KEGG module M00149), and second-carbon oxidation (KEGG module M00311) reflecting perturbed central carbon metabolism (Fig. S3A). In addition, depletion in the mevalonate (MVA) pathway for isoprenoid biosynthesis (KEGG module M00095) was also associated with fluoride treatment. 3-Hydroxy-3-methylglutaryl-coenzyme A reductase, a key enzyme in the MVA pathway, has previously been shown to be fluoride sensitive (26, 27). Carriage of the MVA pathway is limited to Gram-positive taxa such as *Streptococcus*, *Lactobacillus*, and *Staphylococcus* (28). Individually, these taxa were not found to be significantly depleted in fluoride-treated communities via multivariate analysis (Table S2). This analysis suggests that the changes reflected in differentially abundant oral functional modules may result from cumulative small changes in microbial community composition rather than large changes in specific taxa.

10.1128/mSystems.00047-17.3 FIG S3  Bacterial functional modules and genes associated with energy production are affected by fluoride in the oral microbial community. (A) Statistically significant functional modules that are consistently depleted in low- and high-fluoride treatment groups compared to the control group. Gene families were inferred from 16S-based taxonomic profiles using PiCRUSt, and functional modules were reconstructed using HUMAnN (25). (B) Summary of possible fluoride inhibitory mechanism on microbial carbohydrate metabolism. (C) PICRUSt accuracy relative to NSTI scores colored by site (oral and stool). (D) Scatterplots illustrate the correlation between KO relative abundance that was measured (by shotgun metagenomics sequencing using HUMAnN) (*y* axis) and KO relative abundance that was predicted (by PICRUSt analysis of 16S) (*x* axis). Median stool (left) and oral (right) samples are shown. (E) Rarefaction curve illustrating the number of KEGG orthogroups (KOs) (*y* axis) identified as a function of the number of reads of each sample (*x* axis). Eighty-nine percent of the samples were saturating with respect to KO richness, defined here as a <10% increase in detected KOs after doubling sequencing depth. Download FIG S3, PDF file, 0.6 MB. Copyright © 2017 Yasuda et al. 2017 Yasuda et al. This content is distributed under the terms of the Creative Commons Attribution 4.0 International license.

10.1128/mSystems.00047-17.7 TABLE S3  Fluoride treatment effects on oral bacterial functional modules at week 12. Download TABLE S3, XLS file, 0.02 MB. Copyright © 2017 Yasuda et al. 2017 Yasuda et al. This content is distributed under the terms of the Creative Commons Attribution 4.0 International license.

**FIG 3:**
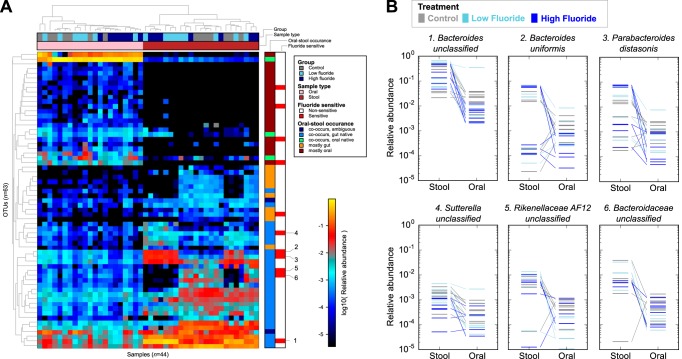
Fluoride affects stool-derived taxa found in the oral cavity. (A) Sixty-three abundant OTUs (rows) across oral and stool samples (columns). Rows and columns are clustered by Bray-Curtis dissimilarity. OTUs are shown in colors according to their biogeographic occurrence/cooccurrence patterns (see main text for definitions). OTUs that were significantly depleted in fluoride-treated oral samples are shown in red, including a subset of orally abundant, stool-derived OTUs. The six OTUs in this subset with the greatest treatment effects are highlighted in panel B. Horizontal lines represent individual relative abundance measurements (colored by treatment group), and measurements from the same animal are connected.

The quality of the predicted functional profiles analyzed above can be estimated from the nearest sequenced taxon index (NSTI), which measures the closeness of a 16S-based profile to known reference genomes. NSTI values for our samples were low (i.e., close to reference), and mean NSTI scores for oral and gut samples were 0.047 and 0.148, respectively (Table S4 and Fig. S3C). This range of NSTI values (i) is consistent with other nonhuman mammal-associated samples and (ii) suggests that predicted versus measured functional profiles for these samples should be in reasonably strong agreement (see Fig. 3 in reference 24). This agreement was also directly measured by subjecting a subset of samples to shotgun metagenomic sequencing and profiling (described in a subsequent section).

10.1128/mSystems.00047-17.8 TABLE S4  Genus-level taxonomic profiles of all samples from 16S sequencing and their associated metadata. Download TABLE S4, XLS file, 0.3 MB. Copyright © 2017 Yasuda et al. 2017 Yasuda et al. This content is distributed under the terms of the Creative Commons Attribution 4.0 International license.

### Fluoride affects stool-derived microbes in the mouse oral microbial community.

We hypothesized above that the lack of fluoride treatment effect in the gut could be due to the low concentration of fluoride reaching that environment. In principle, gut microbes exposed to fluoride in the oral cavity (where concentrations are expected to be higher during treatment) might reveal additional sensitivity. While oral and gut microbial taxa are largely distinct in humans (15), mice are coprophagic, and hence have much greater potential for cooccurrence of oral and gut microbes (thus providing a basis to test this hypothesis).

We began by dividing mouse operational taxonomic units (OTUs) according to their biogeographic occurrence patterns (Fig. 3A and Fig. S4). We focused on OTUs that were confidently detected among the week 12 oral or gut samples, defined as having a relative abundance of >10^−4^ (0.01%) in at least five samples from at least one body site. OTUs that were confidently detected in only a single site were classified as “mostly oral” (*n* = 21) and “mostly gut” (*n* = 10). Thirty-two additional OTUs were confidently detected at both sites. We further divided these OTUs into groups based on their likely point of origin. “Cooccurring, oral native” OTUs (*n* = 3) were defined to have mean oral abundance of more than twofold higher than the mean gut abundance and may cooccur as a result of oral-gut transit. Conversely, 27 OTUs were classified as “cooccurring, gut native” due to more than twofold higher mean abundance in the gut (the two remaining cooccurring OTUs were classified as having an “ambiguous” point of origin). Hence, oral sites are colonized by a relatively large number of gut bacterial taxa in mice, which is likely the result of direct or indirect ingestion of stool (with indirect ingestion of stool including grooming of stool-contaminated body parts).

10.1128/mSystems.00047-17.4 FIG S4  Correlation of bacterial OTUs found in both the oral site and stool. Each dot corresponds to the mean relative abundance of an OTU across seven mice for oral (*y* axis) and stool (*x* axis) samples. Marks on the *x* axis (vertical lines) or *y* axis (horizontal lines) margins represent OTUs with zero measured abundance at one site but nonzero abundance at the other. *r*_*s*_, Spearman correlation. Download FIG S4, PDF file, 0.1 MB. Copyright © 2017 Yasuda et al. 2017 Yasuda et al. This content is distributed under the terms of the Creative Commons Attribution 4.0 International license.

We next reexamined the behavior of the 27 orally occurring, stool-derived taxa upon fluoride treatment based on the modeling results described above. While none of these taxa were significantly perturbed in the analysis of stool data, six were differentially abundant between week 12 control and low- or high-fluoride treatment oral samples. The most abundant of these taxa include *Parabacteroides distasonis*, *Bacteroides uniformis*, and unclassified species of *Bacteroides* (Fig. 3B). In fact, the taxa that were depleted among fluoride-treated oral samples were weakly enriched for taxa derived from the stool (*P* = 0.034 by two-tailed Fisher exact test). This suggests that species native to the mouse stool microbiota are indeed sensitive to fluoride in concentrations typical of fluoridated water or dental products. However, these taxa are likely protected from fluoride exposure in stool due to the absorption of fluoride in the stomach and small intestine.

### Targeted metagenomic sequencing supports 16S-based conclusions.

In addition to the 16S-based sequencing profiles introduced above, we assayed subsets of oral (*n* = 6) and stool (*n* = 11) samples by shotgun metagenomic sequencing and profiled them with MetaPhlAn2 (29) (for microbial taxonomy) and HUMAnN2 (for gene families and pathways). While the shotgun-sequenced subset was too small for independent, well-powered statistical analysis, it proved useful for supporting our 16S-based findings and for boosting taxonomic and functional resolution.

To facilitate shotgun 16S comparisons, all taxonomic features were collapsed to the family level. Of the 32 microbial families detected by reference-based shotgun metagenomic profiling, all were detected among the 16S profiles. Thirty-seven additional families were seen only in the 16S data, including several families that are uniquely enriched in the mouse (*Turicibacteraceae* and *Odoribacteraceae* [30]; Table S5). This result underscores the utility of 16S-based taxonomic profiling in this study for detecting and quantifying clades that are underrepresented in isolate genome catalogs.

10.1128/mSystems.00047-17.9 TABLE S5  Taxonomic profiles (MetaPhlAn2) for the subset of samples analyzed with shotgun sequencing and their associated metadata. Download TABLE S5, XLS file, 0.1 MB. Copyright © 2017 Yasuda et al. 2017 Yasuda et al. This content is distributed under the terms of the Creative Commons Attribution 4.0 International license.

Conversely, reference-based shotgun metagenomic profiles were advantageous in providing increased taxonomic resolution in our data set. For example, unclassified species of *Streptococcus* and *Pasteurellaceae* discovered through 16S sequencing were revealed in the shotgun sequencing data to be *Streptococcus parasanguinis* and *Haemophilus parainfluenzae* (Table S5). While we detected no major fungal community members by shotgun sequencing, several viral species were detected (fungi and viruses are notably invisible to 16S sequencing). Excluding trace viruses (i.e., present in one sample at a relative abundance of <0.01%), the commonly occurring viruses were mouse mammary tumor virus and murine osteosarcoma virus. Because these viruses belong to the family *Retroviridae*, their detection may be due to endogenous copies in the mouse genome that eluded host read depletion during metagenomic quality control (e.g., due to absence or divergence from the mouse reference genome; see Materials and Methods).

In addition to the NSTI-based evaluation of our predicted functional profiles, we directly compared the predicted versus measured functional profiles for the subset of samples subjected to both 16S and shotgun metagenomic sequencing. Predicted versus measured gene family abundance (KEGG orthogroups [KOs]) were reasonably concordant as measured by Spearman correlation, which ranged from 0.45 to 0.66 (Fig. S3). Notably, these values are in line with the expected agreement between predicted and measured KO abundance profiles inferred from NSTI scores (see Fig. 3 in reference 24), which lends further support to the accuracy of all predicted functional profiles considered in this study.

While most shotgun metagenomes were saturated with respect to their measured functional richness (Fig. S3), undersampling of low-abundance KOs could in principle exaggerate the apparent disagreement between inferred and measured functional profiles. However, the Spearman correlations cited above should be reasonably robust to such events, meaning that any such exaggeration would be small. We suspect that disagreements between our 16S-inferred and metagenomic functional profiles are largely driven by the dependence of inferred profiles on a (complete) microbial reference genome catalog, which may be missing ideal representatives for certain mouse-associated species (consistent with the low but nonzero NSTI scores described above).

## DISCUSSION

Our study surveyed the effect of chronic fluoride intake on oral and gut mouse microbial communities. Specifically, using a combination of 16S rRNA gene and shotgun metagenomic sequencing, we profiled changes in the taxonomic and functional composition of oral and gut communities following exposure to fluoride treatment. Our data revealed that fluoride exposures at levels commonly found in municipal water and dental products induced statistically significant changes in oral, but not stool, microbial community structure and function. In the mouse models used here, microbes in the oral community that are more typical of the gut microbiome (and likely derived from coprophagy) were also selectively depleted by fluoride treatment.

Due to its extremely widespread use in public health, it is important to understand how fluoride, even at low levels of exposure, might affect human-associated microbial communities during or after chronic use. Fluoride use in humans was first studied in the 1950s by assessing the toxicity of fluoride on different host organ systems (31, 32) and on cultures of select oral pathogens such as *Streptococcus mutans* (6–8). More recently, two 16S sequencing studies of human populations have assessed the effect of fluoride on the dental plaque microbiome (13) (*n* = 12) and orthodontic fixed appliances (14) (*n* = 91). Both studies considered only the oral microbiome and, again, observed only minor, low-effect size shifts in microbial composition longitudinally after fluoride exposure. Neither study included cross-sectional controls lacking fluoride exposure, nor were subjects at baseline free of chronic fluoride exposures from routine dental hygiene. To our knowledge, our work is thus the first study to assess the independent effects of fluoride on the oral and gut microbial communities.

Previous *in vitro* studies have shown that fluoride inhibits a wide variety of enzymes, including phosphatases, pyrophosphatases, esterases, and catalases (33, 34). This inhibition is typically due to interactions with cationic metal cofactors. Among the best-characterized fluoride-sensitive enzymes are those involved in glycolysis (e.g., enolase [35]) and the citric acid cycle (e.g., succinate dehydrogenase [36]). However, the inputs into both of these processes (glucose and pyruvate) can be alternatively metabolized via the hexose monophosphate shunt or fermentation (37) if critical enzymes are inhibited by fluoride. Although enolase is the best-characterized fluoride-sensitive enzyme, it was not depleted in our functional data due to its universal carriage in bacteria (38). Our data show, however, that *in vivo* fluoride-associated modulation of *in vitro*-demonstrated fluoride-sensitive genes (such as succinate hydrogenase and glyoxylate reductase [39]) is detected in metagenomic data. While 16S-based functional predictions cannot associate specific gene polymorphisms with fluoride sensitivity, future studies aimed at specific microbial molecular products or physiology may provide a higher-resolution look at specific genes and activities affected by fluoride.

Our work, along with several previous, more-open-ended studies of human populations (13, 14), suggest that physiological fluoride exposure levels have little effect on the established gut microbiome and even on the overall composition of the oral microbiome. An interesting open question, however, is the degree to which this conclusion might differ in developing microbial communities such as the infant gut or oral microbiota within the first few years of life (40–42). While it is difficult to model early developing human microbial communities in mouse systems, we anticipate that future work with carefully designed models, human populations, or directly investigating microbial physiology will continue to characterize the effects of this daily environmental exposure on microbial community composition and function.

We have shown that exposures to fluoride levels found in municipal water and dental products altered oral, but not stool, microbial community structure and function in mice. Specifically, genera containing acidogenic bacteria, such as *Parabacteroides*, *Bacteroides*, and *Bilophila*, were depleted in the mouth, and fluoride exposure was associated with depletion of genes involved in central carbon metabolism and energy harvest. In contrast, fluoride treatment did not have a significant effect on gut community composition or function, which is consistent with administered fluoride not reaching the gut to an appreciable extent. While the specific responding taxa in humans and in mice are unlikely to be the same due to differences between human systems and mouse models, the mechanisms of response and overall community structural ecological changes are likely to be shared (43). We conclude that in our model, exposure to fluoride levels found in municipal water and dental products had selective effects on the composition of the oral microbial community but are unlikely to have significant effects on established gut microbial communities.

## MATERIALS AND METHODS

### Animal husbandry.

Female BALB/c mice were weaned between postnatal days 18 to 21 and randomized into experimental cages (four cages per treatment; seven mice per treatment) with an adjustment period of handling the animals for 1 week, each cage containing two mice. Mice were fed irradiated standard mouse chow (PicoLab Mouse diet 20 [catalog no. 5058; LabDiet, St. Louis, MO]). All experiments were approved and conducted in accordance with Harvard Medical School Standing Committee on Animals and National Institutes of Health guidelines.

### Sample collection and processing.

Stool samples were collected at weeks 0, 4, 8, and 12. Oral samples were collected at weeks 0 and 12. Since the animals had to be euthanized to collect the oral samples (to avoid mouse skin contamination), our week 0 oral samples were collected from a separate set of mice (*n* = 8) that were cohoused until randomization at week 0. The collected samples were stored at −80°C before processing. DNA was extracted using the MP Bio FastDNA Spin kit for soil (MP Bio, Santa Ana, CA) according to the manufacturer’s instructions. For the 16S rRNA amplicon sequencing, the V4 region was amplified using the Earth Microbiome Project 16S sequencing protocol (44). In brief, genomic DNA was subjected to 16S rRNA gene (16S) amplifications using primers designed incorporating the Illumina adapters and a sample barcode sequence, allowing directional sequencing covering variable region V4 (primers 515F [F stands for forward] [AAT GAT ACG GCG ACC ACC GAG ATC TAC ACT ATG GTA ATT GTG TGC CAG CMG CCG CGG TAA] and 806R [R stands for reverse] [GGACTACHVGGGTWTCTAAT]). PCR mixtures contained 2 μl of diluted template (5 to 50 ng/µl of DNA), 10 μl of HotMasterMix with the HotMaster Taq DNA polymerase (5 Prime), and 0.5 μl of primer mix (10 μM of each primer). The cycling conditions consisted of an initial denaturation of 94°C for 3 min, followed by 32 cycles of denaturation at 94°C for 45 s, annealing at 50°C for 60 s, extension at 72°C for 5 min, and a final extension at 72°C for 10 min. Amplicons were quantified using a Qubit 2.0 fluorometer and Quant-iT PicoGreen double-stranded DNA (dsDNA) assay kit (Invitrogen, Life Technologies). Integrity of DNA was tested by gel electrophoresis (1% agarose gel). Quantified DNA was pooled in equimolar concentrations, size selected (375 to 425 bp) on the Pippin Prep system (Sage Sciences, Beverly, MA) to reduce nonspecific amplification products from host DNA. Sequencing was performed on the Illumina MiSeq platform (version 2) according to the manufacturer’s specifications with the addition of 15% PhiX and yielded paired-end reads of 151 bp in length in each direction.

A subset of samples used for 16S sequencing was subjected to shotgun metagenomic sequencing. This subset includes 6 oral samples (3 control samples and 3 samples from the high-fluoride group at the end of the study) and 11 stool samples (3 control samples and 2 or 3 samples from the high-fluoride group at the beginning and end of the study). Nextera libraries were prepared manually following the manufacturer’s protocol (Nextera XT DNA Sample Prep kit; Illumina Inc., San Diego, CA). Briefly, tagmentation of samples was performed using 1 ng of template, and PCR amplification was performed by a Bio-Rad T100 thermocycler (Bio-Rad, Hercules, CA) following the manufacturer’s protocol. Agencourt AMPure PCR purification system (catalog no. A638801; Beckman Coulter, Brea, CA) was used to select for 300- to 500-bp fragments. The DNA libraries were validated with an Agilent 2100 bioanalyzer (Agilent Technologies, Palo Alto, CA) and quantified using a Qubit2.0 fluorometer (Thermo Fisher Scientific, Waltham, MA). Equal volumes of normalized libraries were combined, diluted in hybridization buffer, and heat denatured, according to Nextera XT protocol. Paired-end sequencing was performed using the NextSeq Mid 150 cycle (2 × 75 bp).

### Bioinformatic analysis of 16S and metagenomic shotgun sequencing.

Overlapping 16S paired-end reads were stitched together (approximately 97-bp overlap) and further processed in a data curation pipeline implemented in QIIME 1.8.0 (QIIME stands for Quantitative Insights Into Microbial Ecology) as pick_closed_reference_otus.py (44). In brief, this pipeline picks operational taxonomic units (OTUs) using a reference-based method and then constructs an OTU table (see Table S4 in the supplemental material). Taxonomy was assigned using the Greengenes (2013 version) predefined taxonomy map of reference sequence OTUs to taxonomy. A mean sequence depth of 134,046 reads per sample was obtained; samples with fewer than 50,000 filtered sequences were excluded from downstream analysis. Further microbial community analyses such as beta diversity calculation and PERMANOVA between variables (i.e., treatment groups, time points, and cages) were performed using the weighted UniFrac distance measure with QIIME 1.8.0 (44). Microbial functional modules were inferred from the 16S-based taxonomic profiles using PICRUSt (Phylogenetic Investigation of Communities by Reconstruction of Unobserved States) (24), and functional modules were reconstructed using HUMAnN (25).

Shotgun metagenomic sequences were first adapter trimmed using cutadapt (45). Mouse reads were removed using KneadData (http://huttenhower.sph.harvard.edu/kneaddata), which also trimmed low-quality base pairs (Phred score < 20) and filtered short reads (trimmed length of <70% of original length). Mouse reads were matched against the BALB/c genome dated February 2017 (http://www.sanger.ac.uk/science/data/mouse-genomes-project). We performed taxonomic profiling using MetaPhlAn2 (29). Species abundances (63 before filtering) were passed through a filter requiring each species to have at least 0.01% abundance in at least 10% of all samples, resulting in 36 species for analysis (Table S5). Functional profiles were generated using HUMAnN2 (25) (http://huttenhower.sph.harvard.edu/humann2). The UniRef90 database (46) was used for the translated search. UniRef90 abundances were collapsed to KEGG Orthology (KO) groups (47) for comparison with PICRUSt output by mapping through UniProt-derived annotations. KO rarefaction analysis (Fig. S3E) was carried out using the rarecurve function in R’s vegan package (step set to 2,500) by providing per-sample KO and unmapped read counts as input.

To test for statistically significant microbial clades associated with fluoride treatment and metadata, we used the combination of LEfSe (17) for univariate and MaAsLin (Multivariate Associations by Linear models) (21) for multivariate analyses. To find taxa enriched in the oral and stool samples, we used LEfSe, where classes are set as biogeographical locations (oral and stool). To identify taxa and functional modules associated with fluoride treatment in each oral sample and stool sample, we used MaAsLin to build a multivariate linear model combining fixed and random effects on each sample type. For the oral samples, week 12 samples were used to identify taxa and functions that were perturbed by fluoride treatment group (control, low fluoride, or high fluoride). For the stool samples, fluoride’s effects on taxa and functions were first tested on the combination of week 4, week 8, and week 12 samples, as these were the time windows that would allow us to observe potential treatment effects. In this model, animal age (week) and housing cage were included as covariates. We conducted a separate series of MaAsLin in analyses stratifying the samples by week and including housing cage as the only covariate. Across linear models, we applied Benjamini-Hochberg multiple testing correction with a target false-discovery rate (FDR) of 0.2.

### Determining fluoride concentrations for use in mice.

Mice in our study were treated with fluoride by inclusion in their drinking water (low- and high-fluoride groups) and through additional gavage (high-fluoride group). The fluoride concentrations in drinking water were designed to reflect human-equivalent exposures. Specifically, we prepared water with 4 ppm fluoride using sodium fluoride (solubility >99%; catalog no. S6776-100G, Sigma-Aldrich). This is the highest FDA-approved level of fluoride in municipal water in the United States. Mice in the low- and high-fluoride groups drank from this solution daily throughout the experiment. This is equivalent to a 0.02-µg/day exposure, based on an expected consumption of 5 ml of solution per day (48).

Mice in the high-fluoride group received an additional dose of 2.25 µg of fluoride per day. This dose was based on equivalent amounts of fluoride that might be consumed by ingestion of fluoridated toothpastes in young children (1 to 3 years of age). Specifically, we assumed a 10-kg child consuming one-quarter of 1 g of toothpaste per brushing session, twice per day, with a typical toothpaste fluoride concentration of 1,500 µg per gram. This equates to 750 µg per day for the 10-kg child, which is equivalent to 2.25 µg per day for a 30-g mouse (the expected average mass of our mice over the 12-week time course). This additional fluoride was given daily to mice in the high-fluoride group by gavage in 10 μl of deionized water.

### Measuring fluoride concentrations in the intestinal contents.

We measured fluoride ion concentrations in mouse stool samples using a fluoride ion electrode probe (Cole-Parmer Instrumental Company, Vernon Hills, IL). We calibrated the probe using an initial 1,000 ppm fluoride solution provided by the manufacturer serially diluted to 0.001 ppm in deionized water. To analyze a given stool sample, 100 mg of dry stool was dissolved in 10 ml of deionized water. On the basis of the calibration curve, we concluded that untreated mouse stool had a baseline fluoride ion concentration of 0.064 ± 0.032 µg/ml of dissolved stool (mean ± standard deviation [SD], week 0 mice; Fig. S1). In comparison, stool fluoride concentrations after 12 weeks of low-fluoride treatment were not appreciably larger than untreated values at week 0 (0.036 ± 0.013 µg/ml of dissolved stool; *P* = 0.997 by one-tailed *t* test). Fluoride concentrations in stool samples from the high-fluoride group were similarly unaffected (0.065 ± 0.023 µg/ml of dissolved stool; *P* = 0.820).

To confirm that the probe was able to detect additional fluoride in stool, we mixed untreated dissolved stool samples with increasing concentrations of fluoride. This produced a trend similar to the standard calibration curve but flattened below 0.15 µg/ml of dissolved stool (consistent with the inability to resolve concentrations of fluoride below the baseline measurement for mouse stool). A follow-up experiment using stool subjected to bead beating (to potentially release additional intracellular fluoride) produced a similar trend.

### Accession number(s).

Data needed to evaluate the conclusions in the paper are present in the paper and supplemental material, and the sequences generated in this study are publicly available (NCBI BioProject identification [ID] or accession number PRJNA328099).
