# Supplementary figures and images for: Fluoride Depletes Acidogenic Taxa in Oral but Not Gut Microbial Communities in Mice
Source: mSystems. 2017 Aug 8;2(4):e00047-17. doi: 10.1128/mSystems.00047-17 (PMC5547758; doi:10.1128/mSystems.00047-17)

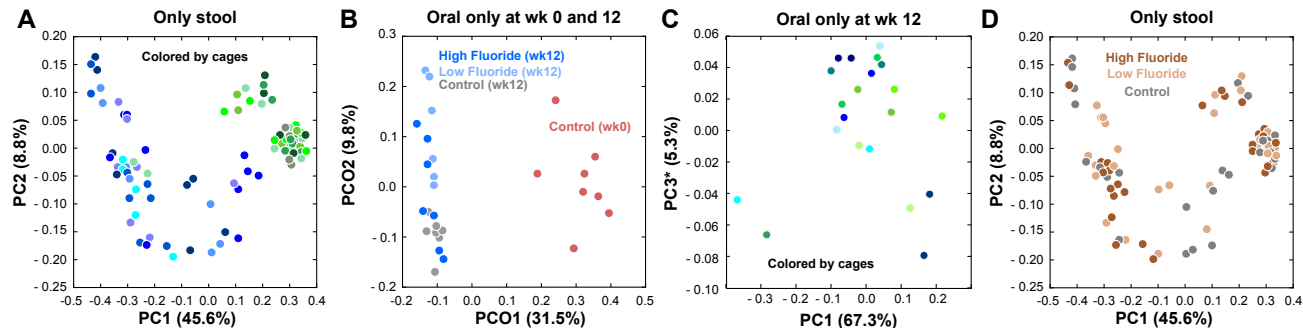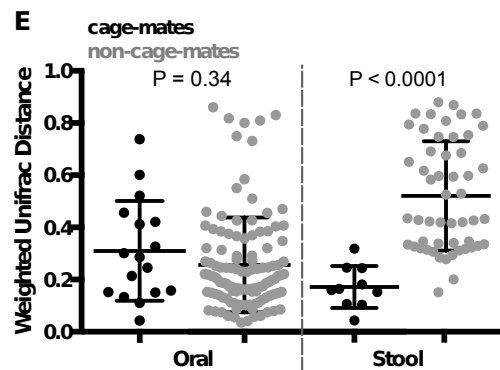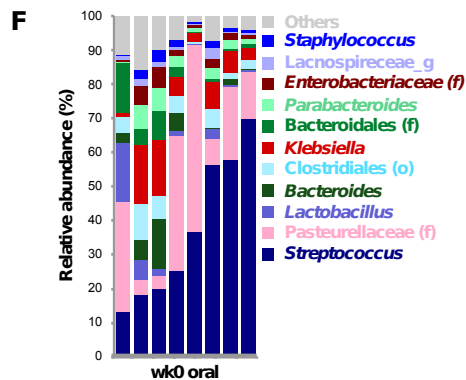

**G** Stool microbiota composition at weeks 0, 4, 8, and 12 (genus)

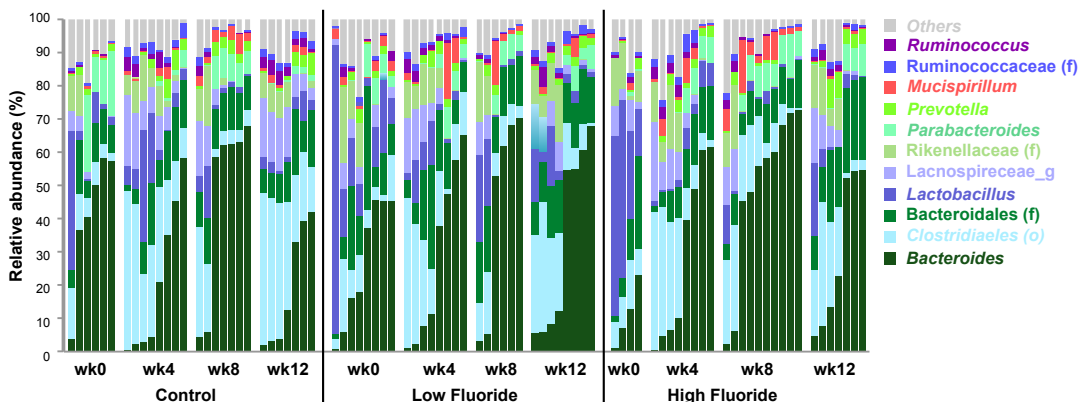

Supplement: FIG S1 [file sys004172123sf1.pdf]

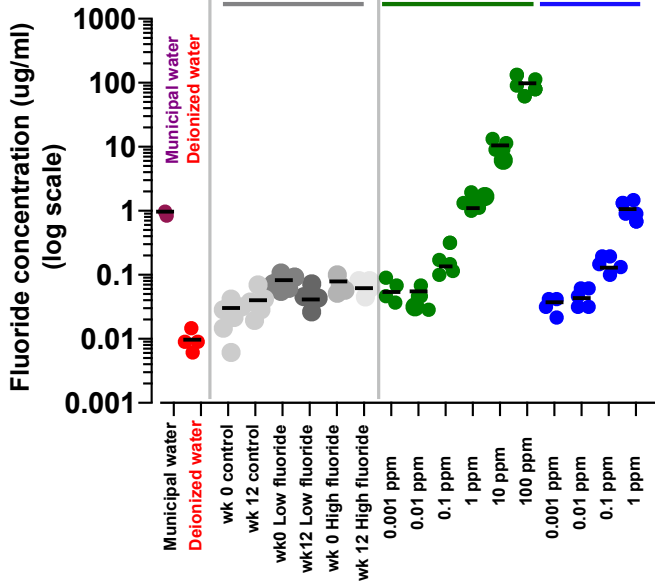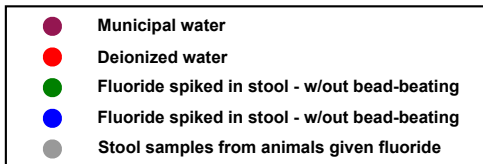

Supplement: FIG S2 [file sys004172123sf2.pdf]

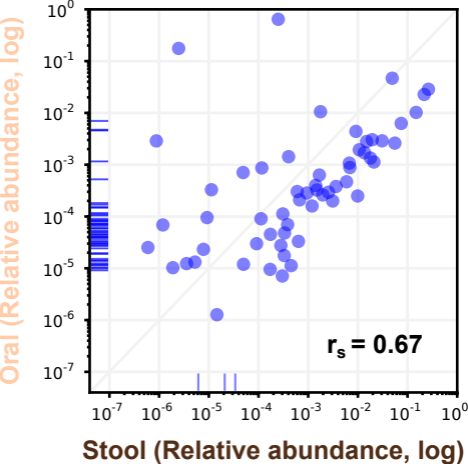

Supplement: FIG S4 [file sys004172123sf4.pdf]
