# Supplementary material for: Fluoride Depletes Acidogenic Taxa in Oral but Not Gut Microbial Communities in Mice
Source: mSystems. 2017 Aug 8;2(4):e00047-17. doi: 10.1128/mSystems.00047-17 (PMC5547758; doi:10.1128/mSystems.00047-17)
Supplement: FIG S3 [file sys004172123sf3.pdf]

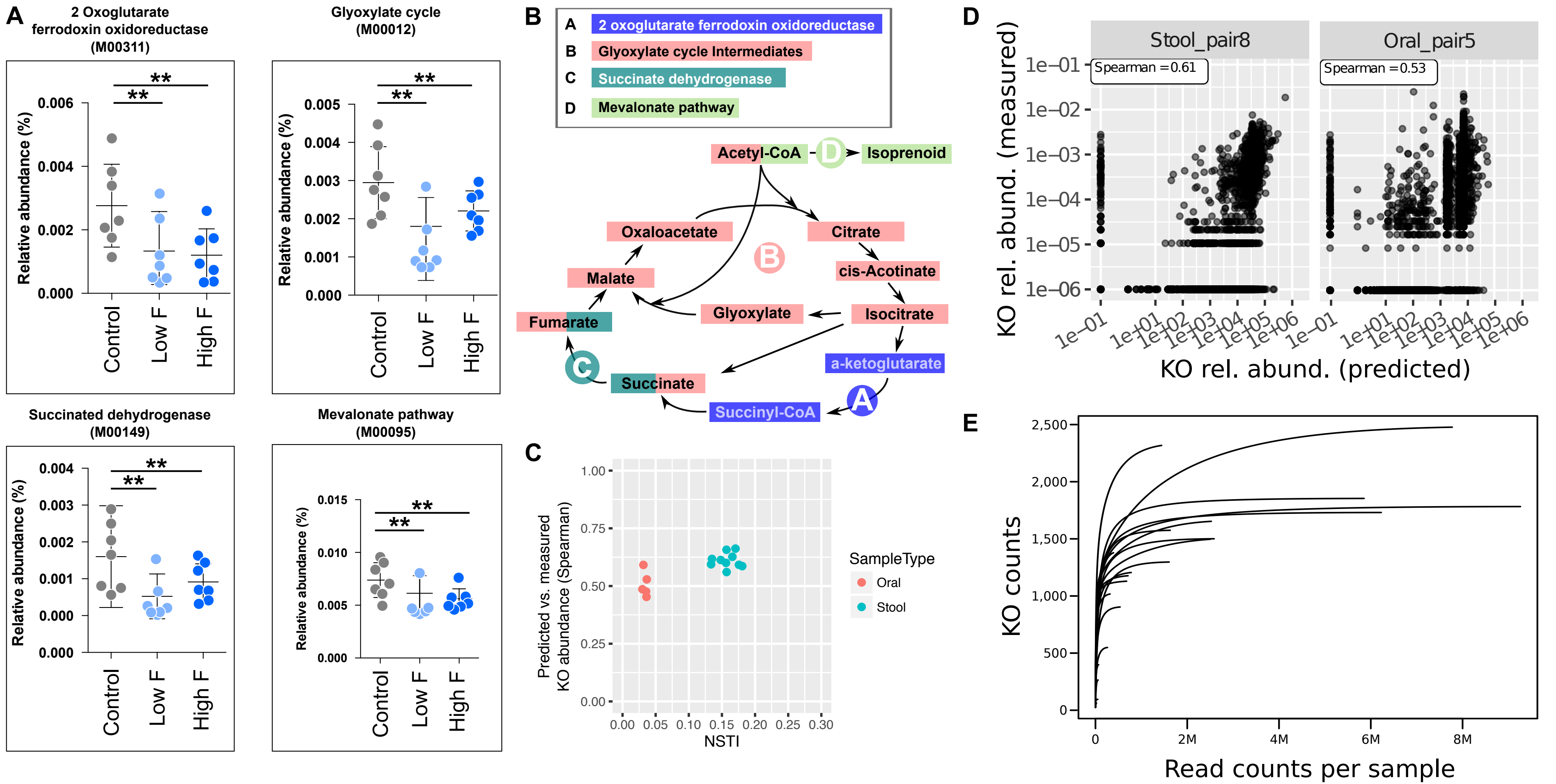
A
2 Oxoglutarate ferrodoxin oxidoreductase (M00311)
Glyoxylate cycle (M00012)
Succinated dehydrogenase (M00149)
Mevalonate pathway (M00095)
Relative abundance (%)
Control
Low F
High F
**
B
A 2 oxoglutarate ferrodoxin oxidoreductase
B Glyoxylate cycle Intermediates
C Succinate dehydrogenase
D Mevalonate pathway
Acetyl-CoA
Isoprenoid
Oxaloacetate
Citrate
cis-Acotinate
Isocitrate
Glyoxylate
Malate
Fumarate
Succinate
Succinyl-CoA
a-ketoglutarate
C
Predicted vs. measured KO abundance (Spearman)
NSTI
SampleType
Oral
Stool
D
Stool_pair8
Oral_pair5
Spearman =0.61
Spearman =0.53
KO rel. abund. (measured)
KO rel. abund. (predicted)
E
KO counts
Read counts per sample
